# The Efficacy of Cladribine (2-CdA) in Advanced Systemic Mastocytosis

**DOI:** 10.1007/s12288-020-01279-8

**Published:** 2020-04-15

**Authors:** Grzegorz Helbig, Anna Koclęga, Władysław B. Gaweł, Martyna Włodarczyk, Marek Rodzaj, Anna Łabędź, Iwona Hus, Małgorzata Raźny

**Affiliations:** 1 Department of Hematology and Bone Marrow Transplantation, School of Medicine in Katowice, Medical University of Silesia, Dąbrowski street 25, 40-032 Katowice, Poland; 2 Department of Hematology and Bone Marrow Transplantation, Students’ Research Group, Katowice, Poland; 3 Department of Hematology and Internal Medicine, Rydygier Hospital, Kraków, Poland; 4 Institute of Hematology and Transfusion Medicine, Warsaw, Poland

**Keywords:** Aggressive systemic mastocytosis, Cladribine, Systemic mastocytosis with an associated hematological neoplasm, *KIT*D816V, Response

## Abstract

Systemic mastocytosis (SM) is a rare clonal disorder with multi-organ involvements and shortened life expectancy. To date, no curative treatment for SM exists. Cladribine (2-CdA) is a purine analogue showing activity against neoplastic mast cells and its use was found to be effective in some patients with SM. Nine patients (six males and three females) with advanced SM at median age of 63 years (range 33–67) who received at least one course of 2-CdA were included in a retrospective analysis. Study patients were classified as having aggressive SM (ASM; *n* = 7) and SM with an associated hematological neoplasm (SM-AHN; *n* = 2). The “C” findings were as follows: (1) absolute neutrophil count (ANC) < 1 × 10^9^/l (*n* = 1) and/or hemoglobin level < 10 g/dl (*n* = 4) and/or platelet count < 100 × 10^9^/l (*n* = 4); (2) hepatomegaly with ascites (*n* = 4); (3) skeletal involvement (*n* = 2); (4) palpable splenomegaly with hypersplenism (*n* = 3) and (5) malabsorption with weight loss (*n* = 5). Treatment consisted of 2-CdA at dose 0.14 mg/kg/day intravenously over a 2-h infusion for 5 consecutive days. Median dose per cycle was 45 mg (range 35–60). Median number of cycles was 6 (range 1–7). Overall response rate (ORR) was 66% (6/9 pts) including three partial responses and three clinical improvements. ORR was 100% and 66% for SM-AHN and ASM, respectively. Median duration of response was 1.98 years (range 0.2–11.2). At the last contact, five patients died, four have little disease activity, but remain treatment- free. 2-CdA seems to be beneficial in some patients with SM, however the response is incomplete.

## Introduction

Systemic mastocytosis (SM) is a rare and complex disease resulting from a clonal proliferation of abnormal mast cells which may accumulate in various organs. Clinical manifestations are variable ranging from pure cutaneous involvement (usually in childhood) to aggressive neoplasm with multi-organ dysfunction and shortened survival (in adults). According to the criteria of World Health Organization (WHO), indolent and advanced variants of SM exist. The indolent form (ISM) is characterized by slow disease course, low mast cell burden and relatively good prognosis. Most patients with ISM do not require cytoreductive therapy. Conversely, the advanced variant has poor prognosis with reduced survival. It includes aggressive SM (ASM), mast cell leukemia (MCL) and SM with an associated hematologic neoplasm (SM-AHN). Patients within this category have at least one “C” finding which reflects tissue damage by mast cells and they require cytoreductive therapy to decrease or reverse organ dysfunction [3, 9, 12, 14].

The diagnosis of SM can be made using WHO criteria. Briefly, the diagnostic procedure starts with bone marrow biopsy or other extra-cutaneous organs. The other tests include mast cell immunophenotyping, a molecular study towards the presence of the *KIT*D816V mutation and the measurement of serum tryptase levels [9].

Cytoreductive therapy is recommended for patients with advanced SM (advSM) due to the frequent presence of life-threatening organ dysfunction and shortened life expectancy. The therapeutic approach for SM-AHN is especially challenging as one has to decide which disease component (AHN or SM) requires more immediate treatment [6].

An off-label use of cladribine (2-CdA) remains a mainstay of therapy for advSM. Cladribine is a purine analogue that shows in vitro and in vivo inhibitory effect on neoplastic mast cells. Its mechanism of action does not involve tyrosine kinase inhibition therefore it causes apoptosis in mast cells independently of the presence of the *KIT*816V mutation. The drug is generally well tolerated, however its use may cause prolonged myelo- and immunosuppression with subsequent occurrence of opportunistic infections [5].

Herein, we report on our experience with cladribine in nine patients with advSM.

## Material

### Patients and Methods

The study patients were identified through the use of our institutional database of medical records. The diagnosis of SM was based on criteria outlined in the 2008 World Health Organization (WHO) document [9]. Trephine biopsy with tryptase, CD25 and CD117 immunostaining as well as immunophenotyping studies using monoclonal antibodies against CD2/CD25/CD117 antigens were performed on bone marrow cells. Serum tryptase levels were assessed using Uni-CAP-Tryptase fluoroimmunoassay (Pharmacia & Upjohn, Uppsala, Sweden; *N* < 11.4 ng/ml). The abovementioned tests were carried out before 2-CdA initiation and then repeated after treatment completion for response assessment. Identification of the *KIT*D816V mutation was done on peripheral blood or bone marrow cells using allele-specific polymerase chain reaction (PCR). Data on cytogenetic results were reported only for three patients as dry aspirates were present in the remaining thus metaphases were not obtained. Data were collected between 2008 and 2018. All patients provided an informed consent in accordance with the Declaration of Helsinki.

### Treatment

The main indication for treatment was the presence of at least one C-finding [1]. Treatment consisted of 2-CdA (Biodribin, Institute for Biotechnology and Antibiotics, Warsaw, Poland) at dose 0.14 mg/kg/day intravenously over a 2-h infusion for 5 consecutive days. The cycles were repeated every 4–6 weeks depending on decision of treating physician. All patients received prophylactically co-trimoxazole, fluconazole and acyclovir.

### Response Assessment

Response assessments were performed after completion of three and six cycles of 2-CdA and were based on consensus criteria proposed by International Working Group-Myeloproliferative Neoplasms Research and Treatment (IWG-MRT) & European Competence Network on Mastocytosis (ECNM) [8].

### Statistics

The probability of overall survival (OS) was estimated using the method of Kaplan and Meier.

## Results

Nine patients (six males and three females) with advSM at median age of 63 years at diagnosis (range 33–67) who received at least one course of 2-CdA were included in a retrospective study. Males were older than females (65 years vs 37 years; *p* = 0.06). The patients were classified as having ASM (*n* = 7) and SM-AHN (*n* = 2). The latter variant depicted myelodysplastic syndrome (MDS) and chronic myelomonocytic leukemia (CMML). Median white blood cell (WBC) and eosinophil counts were within normal range whereas median hemoglobin and platelet counts were lower than normal (9.8 g/dl and 144 × 10^9^/l, respectively). Median alkaline phosphatase level was mildly increased (125 IU/l; normal range 32–92) whereas median albumin level was within normal range (39 g/l; normal range 35–52 g/l). Details were shown in Table 1.

**Table 1:** Characteristic of patients with advanced systemic mastocytosis at study entry

Parameter	Result
Number of patients	9
Gender (F/M)	3/6
Age (median; range)	63 (33–67)
Variant (*n*; %)	
ASM	7
SM-AHN (MDS, CMML)	2
WBC (× 10^9^/l); median, range > 10^9^/l; (*n*) %	11.1 (1.3–41.7) 5 (55)
AEC (× 10^9^/l); median, range > 1.5 × 10^9^/l; (*n*) %	0.22 (0.01–29.2) 3 (33)
Hgb (g/dl); median, range < 10 g/dl (*n*) %	9.8 (6.3–12.5) 5 (55)
PLT (× 10^9^/l); median, range < 100 × 10^9^/l (*n*); %	144 (33–252) 4 (44)
AF (IU/l); median, range > N (*n*); %	125 (56–1030) 8 (88)
Albumin (g/l); median, range < N (*n*); %	39 (28–43) 3 (33)
Serum tryptase (ng/ml); median, range > 200 ng/ml (*n*); %	199 (25.4–446) 4 (44)
Hepatomegaly (%)	77
Splenomegaly(%)	100
Lymphadenopathy (%)	33
Bone lesions (%)	22
Urticaria pigmentosa (%)	55
B symptoms (%)	67
Mast cells in bone marrow (%); median, range	30 (15–80)
Bone marrow cellularity (%); median, range	100 (35–100)
Bone marrow fibrosis (*n*)	
Grade I	3
Grade II	5
Grade III	1
c-kit D816V (%)	67

*AEC* absolute eosinophil count, *AF* alkaline phosphatase, *ASM* aggressive systemic mastocytosis, *CMML* chronic myelomonocytic leukemia, *F* female, *Hgb* hemoglobin, *M* male, *MDS* myelodysplastic syndrome, *PLT* platelets, *SM-AHN* systemic mastocytosis with an associated hematological neoplasm, *WBC* white blood cell

Serum tryptase levels were increased in all study patients; and were above 200 ng/ml in 44% of them. Flow cytometry tests detected the presence of abnormal CD2+/CD25+/CD117+ cells in marrow aspirate in all but two. 67% of the study population demonstrated the presence of the *KIT*D816V mutation. No patient with detectable *FIP1L1-PDGFRA* fusion gene was found. Karyotype was available only for three patients; and was diploid in two and one patient had trisomy of chromosome 8.

The “C” findings being the indication for treatment initiation were as follows: (1) absolute neutrophil count (ANC) < 1 × 10^9^/l (*n* = 1) and/or hemoglobin level < 10 g/dl (*n* = 4) and/or platelet count < 100 × 10^9^/l (*n* = 4); (2) hepatomegaly with ascites (*n* = 4); (3) skeletal involvement (*n* = 2); (4) palpable splenomegaly with hypersplenism (*n* = 3) and (5) malabsorption with weight loss (*n* = 5). The most frequent mediators release symptoms included fatigue (62%), pruritus (37%), abdominal pain (37%) and flush (25%). More than 60% of patients had at least one “B” symptom at diagnosis (fever, night sweats, weight loss). On physical examination all study patients demonstrated splenomegaly, whereas hepatomegaly was present in 77% of them. Urticaria pigmentosa was present in 55% of study cases. Three patients had lymphadenopathy.

All patients received 2-CdA intravenously for 5 consecutive days. Median dose per cycle was 45 mg (range 35–60). Median number of cycles was 6 (range 1–7). The interval between cycles was 28 days, except two patients who received only one cycle of 2-CdA (discontinued due to disease progression). In general, treatment with 2-CdA was well tolerated with no grade 3/4 non-hematological adverse events. One patient had fever with upper respiratory tract infection during the first 2-CdA cycle. Blood culture was negative and infection resolved after the treatment with empirical antibiotics. The other patients remained infection-free during the whole therapy. One patient developed asymptomatic grade 4 neutropenia after the first 2-CdA treatment, but it resolved rapidly after five doses of granulocyte colony-stimulating factor (G-CSF). None of the remaining patients had grade 3–4 neutropenia during the treatment, however grade 2 neutropenia was common during the 2-CdA treatment. Only two patients developed grade 2 thrombocytopenia and it remained stable throughout the whole treatment. None of the patients required platelet transfusions.

Overall response rate (ORR) was 66% (6/9 pts) including three partial responses (PR) and three clinical improvements (CI). ORR was 100% and 66% for SM-AHN and ASM, respectively. Median duration of response was 1.98 years (range 0.2–11.2). Among six responders, three patients progressed and two of them eventually died. One patient is alive more than 2 years from 2-CdA completion. Three other responders did not progress, however one of them died due to unclear neurological complications.

Clinical improvements were mainly observed in terms of hematological parameters (increase in ANC and/or hemoglobin and/or platelet count) and hepatosplenomegaly. There was no resolution of skin changes and lymphadenopathy. Mediators release symptoms decreased in most patients. A decrease of serum tryptase levels was observed in all patients. Mast cell infiltration in bone marrow trephine also decreased after therapy, however by less than 50% when compared with treatment initiation. At the last contact, five patients died, four remain alive.

Among expired patients, one underwent allogeneic stem cell transplantation with subsequent steroid-resistant graft versus host disease. The other patients expired due to different causes: unclear neurological complications with paraparesis (*n* = 1), transformation to chemo-resistant acute myeloid leukemia (*n* = 1), progressive mastocytosis (*n* = 2).

Four living patients have little disease activity (PR; *n* = 2 and CI; *n* = 2) and remain treatment-free after median of 2.8 years (range 0.9–11.2) after 2-CdA completion. Table 2.

**Table 2:** Response to treatment with cladribine in advanced systemic mastocytosis

Parameter	Result
Number of cladribine cycles (median, range)	6 (1–7)
Median dose of cladribine per cycle (mg)	45 (35–60)
Response to cladribine (ORR;%)	67
Clinical improvement	3
Partial response	3
Stable disease	1
Disease progression	2
Median duration of response (years); (median, range)	1.45 (0.2–11.2)
Outcome	
Death (*n*;%) due to	5 (56)
Transplant complications	1
Unknown (probably neurological infection)	1
Transformation into acute myeloid leukemia	1
Mastocytosis resistant to therapy	2
Alive (*n*;%)	4 (44)
OS at 1 year (%)	64
OS at 3 years (%)	38

*ORR* overall response rate, *OS* overall survival

## Discussion

The decision whether to treat or not a patient with 2-CdA must be preceded by careful determination of risk-versus-benefit ratio. Nevertheless, it seems reasonable to offer this agent for patients with advanced SM in whom survival is reduced due to multi-organ involvements [1]. The first report on the efficacy of 2-CdA in a patient with advSM resistant to interferon α has been published by Tefferi et al*.* almost 20 years ago. The treatment resulted in complete resolution of skin changes and marked reduction of mast-cell infiltration in bone marrow [13]. Since that time several study groups presented their data on efficacy and safety of 2-CdA, however different variants of mastocytosis were evaluated. In fact, when we narrow the analysis only to patients with advSM, the number of patients treated with 2-CdA was relatively small. Following the successful Tefferi’s case report [13], ten patients with SM received 2-CdA in a Kluin-Nelemans study [10], but only six of them met the criteria of advanced disease (either SM-AHN or ASM). Nevertheless, all patients responded to cytoreductive therapy in terms of organ involvements, hematological and biochemical parameters as well as mediator-release symptoms, however no patient achieved complete remission. The time to best response took several months, but response duration was not provided. Side effects resulted mainly from bone marrow suppression and were manageable. Sixteen patients with advanced SM received 2-CdA in Mayo Clinic with ORR rate of 50% and 55% in ASM and SM-AHN, respectively. Median duration of response was 11 months. The presence of circulating immature progenitors was associated with inferior response in multivariate analysis [11]. The largest series to date on 2-CdA in mastocytosis has been reported by French Group. The study evaluated 68 patients (38 with ISM and 32 with advanced SM). Patients received median of four courses of 2-CdA with ORR of 50% for those with advanced disease (ASM-43% and SM-AHN-59%). Of note is that complete responses have not been achieved. Response duration exceeded 2 years and side effects were typical of those for 2-CdA [4].

Our results seem to confirm those of other study groups. Of note is that 2-CdA dose within all studies was uniform and side effects were typical of this agent. Nevertheless, the major concern of 2-CdA use is related to immunosuppressive and myelosuppressive toxicity. Almost 50% of the French patients developed grade 3 or 4 neutropenia after at least one course of 2-CdA. Moreover, 83% had grade 3/4 lymphopenia. One patient required allogeneic stem cell transplantation due to prolonged pancytopenia. Infectious complications were demonstrated for 22% during the treatment and 9% of patients developed late opportunistic infections. Two solid tumors were observed after long-term follow-up [4]. A fatal case of progressive multifocal leukoencephalopathy (PML) after 2-CdA for mastocytosis as a consequence of prolonged lymphopenia has been recently published [2].

Overall response rates were comparable between our study and those presented by others [4, 10, 11]. Of note is that different response criteria have been used. ORR for the entire cohort was 66%, with ORR of 100% for SM-AHN and 66% for ASM. However, it should be mentioned that only one patient met PR criteria, and no patient achieved CR. Most patients demonstrated clinical improvements with relatively short-term duration. Death rate exceeded 50% after 2 years of follow-up. Interestingly, one patient transformed to chemo-resistant acute myeloid leukemia shortly after 2-CdA. One patient died from unclear neurological complications (PML?), but detailed diagnosis was not performed. In contrary to other studies [4, 10, 11] we did not observe grade 3 or 4 side effects on 2-CdA.

Of note is, that the *KIT*D816V mutation was detected in 67% of our examined patients and all those patients were tested from bone marrow sample. The results of molecular assays were negative when peripheral blood was used for PCR. Unfortunately, due to respective nature of the study, molecular tests on bone marrow samples were not repeated.

In conclusion, 2-CdA seems to be beneficial in some patients with advSM, however the response is incomplete and transient. No complete responses have been demonstrated so far. The recent approval of tyrosine kinase inhibitor-midostaurin may change our management with patients with advM [7]. Nevertheless, ORR was found to be similar to 2-CdA, but head-to-heat study was not performed. The value of maintenance therapy or combination of 2-CdA with midostaurin require further studies.
